# The Burden of Trachoma in Ayod County of Southern Sudan

**DOI:** 10.1371/journal.pntd.0000299

**Published:** 2008-09-24

**Authors:** Jonathan D. King, Jeremiah Ngondi, Gideon Gatpan, Ben Lopidia, Steve Becknell, Paul M. Emerson

**Affiliations:** 1 The Carter Center, Atlanta, Georgia, United States of America; 2 Department of Public Health and Primary Care, University of Cambridge, Cambridge, United Kingdom; 3 The Carter Center, Juba, Southern Sudan; University of California San Francisco, United States of America

## Abstract

**Background:**

Blindness due to trachoma is avoidable through Surgery, Antibiotics, Facial hygiene and Environmental improvements (SAFE). Recent surveys have shown trachoma to be a serious cause of blindness in Southern Sudan. We conducted this survey in Ayod County of Jonglei State to estimate the need for intervention activities to eliminate blinding trachoma.

**Methodology and Findings:**

A cross-sectional two-stage cluster random survey was conducted in November 2006. All residents of selected households were clinically assessed for trachoma using the World Health Organization (WHO) simplified grading scheme. A total of 2,335 people from 392 households were examined, of whom 1,107 were over 14 years of age. Prevalence of signs of active trachoma in children 1–9 years of age was: trachomatous inflammation follicular (TF) = 80.1% (95% confidence interval [CI], 73.9–86.3); trachomatous inflammation intense (TI) = 60.7% (95% CI, 54.6–66.8); and TF and/or TI (active trachoma) = 88.3% (95% CI, 83.7–92.9). Prevalence of trachomatous trichiasis (TT) was 14.6% (95% CI, 10.9–18.3) in adults over 14 years of age; 2.9% (95% CI, 0.4–5.3) in children 1–14 years of age; and 8.4% (95% CI, 5.5–11.3) overall. The prevalence of corneal opacity in persons over 14 years of age with TT was 6.4% (95% CI, 4.5–8.3). No statistically significant difference was observed in the prevalence of trachoma signs between genders. Trachoma affected almost all households surveyed: 384/392 (98.0%) had at least one person with active trachoma and 130 (33.2%) had at least one person with trichiasis.

**Conclusions:**

Trachoma is an unnecessary public health problem in Ayod. The high prevalence of active trachoma and trichiasis confirms the severe burden of blinding trachoma found in other post-conflict areas of Southern Sudan. Based on WHO recommended thresholds, all aspects of the SAFE strategy are indicated to eliminate blinding trachoma in Ayod.

## Author Summary

Trachoma, a neglected tropical disease, is the leading cause of infectious blindness and is targeted for global elimination by the year 2020. We conducted a survey in Ayod County of Jonglei State, Southern Sudan, to determine whether blinding trachoma was a public health problem and to plan interventions to control this disease. We found the burden of trachoma in Ayod to be one of the most severe ever documented. Not only were adults affected by the advanced manifestations of the disease as is typical for older age groups, but young children were also affected. At least one person with clinical signs of trachoma was found in nearly every household, and 1 in 3 households had a person with severe blinding trachoma. Characteristics previously identified as risk factors were ubiquitous among surveyed households, but we were unable to identify why trachoma is so severe in this location. Surgical interventions are needed urgently to improve vision and prevent irreversible blindness in children and adults. Mass antibiotic distribution may alleviate current infections and transmission of trachoma may be reduced if communities adopt the behavior of face washing and safe disposal of human waste. Increasing access to improved water sources may not only improve hygiene but also reduce the spread of guinea worm and other water-borne diseases.

## Introduction

Trachoma is a disease caused by ocular serovars of the bacterial pathogen *Chlamydia trachomatis.* Infection with ocular *C. trachomatis* results in conjunctivitis and clinical signs of active trachoma are distinguished by the presence of follicles on the tarsal conjunctiva and intense inflammation. Repeated infections lead to conjunctival scarring of the upper eye lid which may result in entropion allowing eyelashes to scrape the cornea; a condition referred to as trichiasis. Without corrective lid surgery, lashes continue to abrade the cornea leading to additional infections, ulceration and eventually opacities in the cornea that are irreversible.[1]

Trachoma is the leading cause of preventable blindness worldwide, endemic in 55 countries and most often found in underdeveloped geographic areas within poor populations.[2] The Word Health Organization (WHO) recommends an integrated set of interventions referred to as the SAFE strategy to eliminate blindness due to trachoma.[3] The ‘S’ component involves the promotion and provision of lid surgery to correct trichiasis. Mass antibiotic distribution, the ‘A’ component, is recommended annually for at least three years to reduce current infections in trachoma endemic communities. Facial hygiene (F) through face washing is promoted based on the assumption that washing away infectious ocular and nasal discharge on the face and hands may reduce and prevent transmission of *C. trachomatis.* Finally, the E component entails environmental improvements such as construction and use of latrines to control trachoma-spreading flies that breed in human faeces and provision of accessible water to allow face washing. In 1998 the World Health Assembly called for countries to determine the burden of trachoma, implement the full SAFE strategy in endemic areas and collaborate with the WHO Alliance for the global elimination of blinding trachoma (GET 2020).[4] According to the WHO recommendations, the full SAFE strategy is warranted at the district level if the prevalence of trachomatous inflammation follicular (TF) is greater than 10% in children aged 1–9 years, and trichiasis prevalence in persons aged over 14 years exceeds 1%.[5]

**Figure 1:**
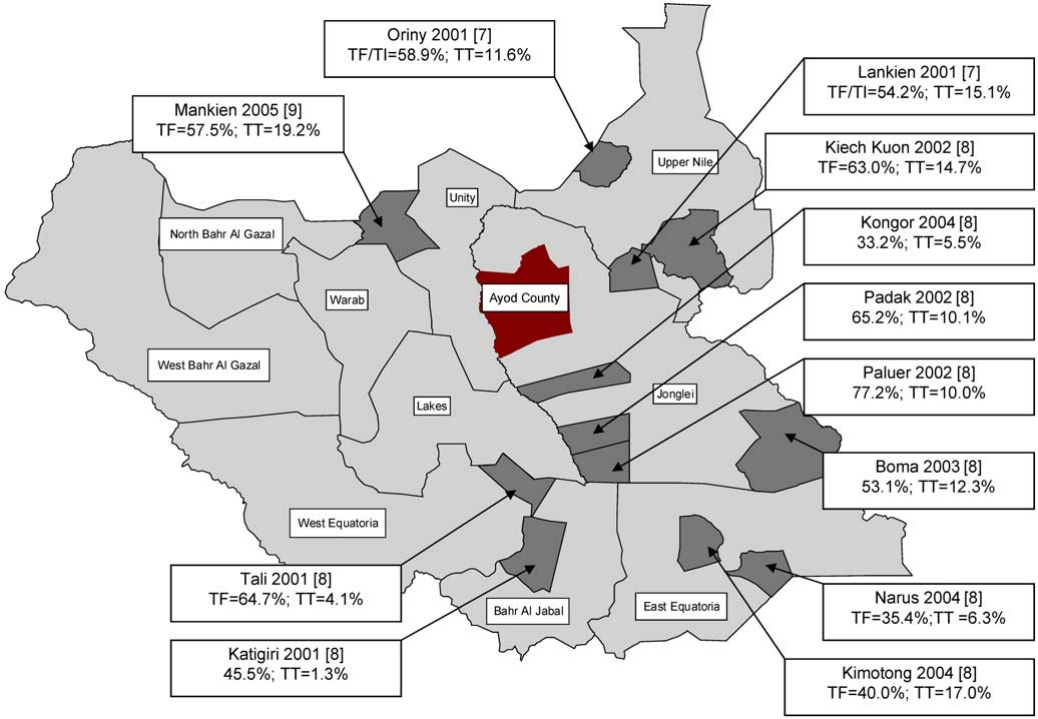
Map of Southern Sudan showing survey site (Ayod County), and areas where previous surveys have been conducted and key prevalence indicators for trachoma. TF, Prevalence of trachomatous inflammation-follicular in children aged 1–9 years; TF/TI, Prevalence of trachomatous inflammation-follicular and or trachomatous inflammation-intense in children aged 1–9 years; TT, Prevalence of trachomatous trichiasis in persons aged 15 years and above.

Many years of civil conflict have impeded development in Southern Sudan resulting in weak infrastructure in most sectors, including health. Additionally, the lack of water and sanitation combine to create an environment conducive not only to trachoma, but also to all the neglected tropical diseases with the exception of Chaga's disease. In particular Southern Sudan has the majority of the current global burden of dracunculiasis (guinea worm disease) and there is a major effort underway to eradicate it.[6] Ayod County is suspected to be endemic for blinding trachoma due to its central location among areas where high burdens of the disease have been documented (Figure 1).[7],[8],[9] We conducted this survey in Ayod County to estimate targets for SAFE intervention activities required to eliminate blinding trachoma. The results of the survey will be used as a baseline reference to monitor and evaluate impact of control interventions on trachoma in Ayod. Secondly, we used the opportunity of interaction with villages to create awareness and expand surveillance for guinea worm disease.

## Methods

### Setting

This survey was conducted in Ayod County shortly after the rainy season from 31 October to 12 November 2006. Ayod is located in the oil-rich northern area of Jonglei State east of the River Nile. The total population is estimated to be 70,000 based on current guinea worm surveillance data.[10] The primary ethnic group in this area of Jonglei is Nuer and the predominant economic activity is farming cattle.

### Sampling

We estimated that a total sample size of 1,650 people of all ages and sexes was required to allow for estimation of at least 50% prevalence of TF in children 1–9 years old within a precision of 10% with a design effect of 5 and a prevalence of 2.5% TT in adults over 14 years old within a precision of 1.5% with a design effect of 2, both at a confidence limit of 95%. We assumed that 29% of the total population was aged 1–9 years of age and 50% were older than 14 years. To achieve the desired sample size we selected twenty clusters of twenty households.

A list of all 202 villages in Ayod County, broken down into seven *payams* (sub-districts), was provided by the Southern Sudan Guinea Worm Eradication Program. Information on individual village population size was not available. A total of 20 villages (clusters) were selected from seven *payams* in the county. Disarmament and sporadic local conflicts were occurring in one payam. For safety reasons, we excluded 27 villages in this payam from the survey. The number of villages selected per payam was proportional to the size of the payam in terms of number of villages. Households within a cluster were selected randomly by using a sketch map and segmentation method as described by Turner et al.[11] This method allows households within a cluster to be selected with equal probability. In addition, these methods allowed us to calculate weights from the selection probabilities to use in the analysis.

### Survey Training

Training of the survey team was performed over a period of seven days by a clinician experienced in trachoma diagnosis, survey methodology, and conducting surveys in Southern Sudan. The survey team consisted of the following persons: 18 guinea worm field officers from Jonglei State; two local health care workers from Ayod village who had received some training in trichiasis surgery; two disease control coordinators; and one international public health professional. Survey workers received training in examination, interviewing, and sketch mapping and segmentation. All team members had prior experience in conducting household surveys and community interventions. Three days of training was conducted in a classroom and four days were utilized for applied training of trachoma grading, data recording, household sampling and interviewing. After the training, members were split into ten teams and transported by air across the county. Teams stayed in central locations near the airstrips where they were dropped and traversed by foot or boat to complete assigned clusters.

### Trachoma grading

**Figure 2:**
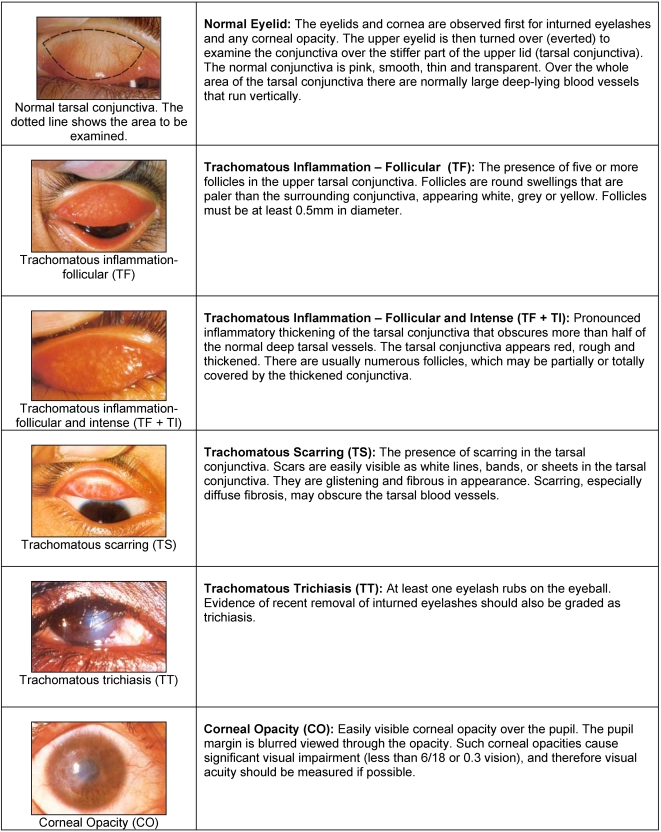
The WHO simplified grading scheme for assessment of Trachoma.

All survey team members received training in the WHO simplified grading system (Figure 2) through explanations, review of slides and field exercises.[12] Each member was required to grade a standardized set of slides to identify clinical signs of trachoma. Only team members achieving more than 80% overall agreement with the trainer's gold standard diagnosis were selected to participate in the field examinations on the fifth day of training. The presence or absence of each sign was assessed separately for each eye of 25 children. After reviewing the individual results of the team members, the trainer spent additional time with examiners on day 6 and day 7 during practical household survey training to confirm diagnoses. In order for a survey worker to be considered an examiner, she/he must have achieved a overall agreement of at least 80% compared to the trainer during the field examinations. Only the best 10 members of the survey team were used as examiners. Examiner's agreement with the gold standard and Cohen's Kappa statistic for each trachoma grade TF,TI and TS, and all three grades combined for the WHO standardized slide set are shown in Table 1.

**Table 1:** Reliability among 10 survey examiners using the WHO standardized set of slides.

Examiner	Inter-observer agreement* (%)			Kappa statistic			
	TF	TI	TS	TF	TI	TS	All**
1	95	93	98	0.9	0.7	0.9	0.9(0.8–1.0)
2	95	93	95	0.9	0.7	0.8	0.9(0.7–1.0)
3	95	93	95	0.9	0.7	0.8	0.8(0.7–1.0)
4	100	83	90	1.0	0.4	0.7	0.8(0.6–0.9)
5	98	85	85	1.0	0.5	0.6	0.7(0.6–0.9)
6	98	85	85	1.0	0.5	0.6	0.7(0.6–0.9)
7	90	75	90	0.8	0.4	0.7	0.7(0.5–0.8)
8	90	78	88	0.8	0.3	0.6	0.6(0.5–0.8)
9	98	88	68	1.0	0.6	0.3	0.6(0.5–0.8)
10	85	68	90	0.7	0.3	0.7	0.6(0.5–0.7)
Kappa for multiple observers				0.8(0.7–0.9)	0.5(0.3–0.5)	0.6(0.04–0.8)	0.7(0.6–0.7)

*: agreement with our gold standard (WHO standardized slides).

**: Combined kappa for grading all three signs (TF, TI and TS).

All residents of each household were enumerated regardless of availability for examination. All eligible residents were examined for trachoma using the WHO simplified grading scheme. Before examining eyes of children, observers determined whether a child's face was unclean. An unclean face was defined as the presence of ocular or nasal discharge. Both eyes were then examined for signs of trachoma using an ×2.5 binocular loupe and adequate light. Each eye was examined for TT and CO prior to everting the eyelid to examine the conjunctiva. Analysis was performed based on the findings of the worst eye. Only cases of CO in the presence of trichiasis were considered trachomatous corneal opacity. Any participant diagnosed with TF or TI was offered tetracycline eye ointment. All participants diagnosed with TT were referred to the health center in Ayod for free trichiasis surgery and follow-up. Survey teams made an effort at the end of the day to examine residents not available during the first household visit.

### Household interview

Using structured questionnaires, heads of household were interviewed by survey team members experienced in conducting health interviews. If the head of household was not available, the acting household decision maker was invited to respond to the interview. Prior to the survey, the questionnaire was translated from English to Nuer and piloted in a village not included in the sample to standardize interviews.

The following characteristics have been shown in previous studies to be potential risk factors for trachoma: absence of a household latrine [9], [13]–[16], residing far from the primary source of water [17]–[21], and disposing of solid waste and keeping cattle near the house.[22]–[24] An increase in reported frequency of face washing and bathing has been associated with a lower occurrence of trachoma in cross sectional studies. [20],[25] To capture information on these potential risk factors, the household interview consisted of questions about: demographics; water source; time to fetch water; practice of face washing; latrine ownership; practice of waste disposal; and cattle keeping. Radio ownership was collected as an indicator of wealth and to determine the feasibility of using radio programming for communication in the future. We considered an improved primary source of water to be a borehole or hand dug well, whilst water from open ponds and dams was considered an unimproved source.

### Data entry and analysis

The data were double entered by two data clerks and compared for consistency using EpiInfo version 3.3.2 (Centers for Disease Control and Prevention [http://www.cdc.gov/EpiInfo/]). Statistical analysis of data was performed in SAS version 9.1 (SAS Institute Inc. [http://www.sas.com/]). Data was weighted according to selection probabilities and stratified by payam. Correlation among the data associated with the complex survey design was accounted for in the estimates and confidence limits by Taylor Expansion using SAS SURVEYFREQ procedures.[26],[27]

Based on the prevalence of clinical signs of trachoma and the household characteristics, we calculated the projected targets for the implementation of the full SAFE strategy in Ayod. The population requiring lid surgery was calculated by multiplying the point estimate and confidence limits of TT prevalence in all age groups by the estimated total population. The target for antibiotic distribution and behavior change communication to promote face washing was determined based on the WHO guidelines for mass treatment where district prevalence of TF in 1–9 year-old children exceeds the threshold of 10%. To calculate the need for environmental improvements, the proportion of households without access to an improved water source and latrines was multiplied by the total number of households in the county to obtain targets for water and sanitation. The confidence limits were used to calculate the lower bounds and upper bounds of the projected targets.

### Ethical considerations

The protocol was reviewed and approved by local government authorities and Emory University Internal Review Board (IRB 079-2006). The purpose of the survey was explained and approval was sought by local authorities in Ayod and village chiefs prior to working in any village. Verbal informed consent to participate in trachoma examination and household interview was obtained from heads of households, each individual and parents of minors according to the principles of the declaration of Helsinki. Documentation of verbal consent was initialed and dated by the examiner on data collection forms as approved by the IRB. Personal identifiers were removed from the dataset prior to analysis.

## Results

### Characteristics of the sample

**Figure 3:**
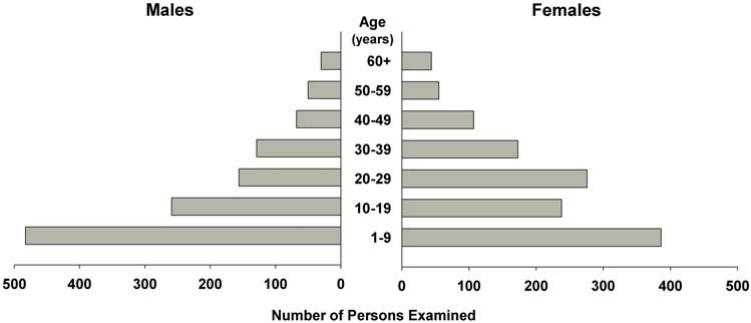
Age Distribution of Survey Population, by sex.

We examined 2,335 participants out of a total of 2,605 residents from 392 households in twenty villages of Ayod County, an 89.6% recruitment rate. All 2,335 participants were examined for TT and CO although lid eversion was not possible on eight participants due to recent trichiasis surgery giving a data set of 2,327 individuals. The mean household size was 7.14 (95% confidence interval [CI], 6.20–8.08) persons per household. Infants less than one year old comprised 3.9% of the examined residents and children 1–9 years of age comprised 36.7%. Persons aged 10 to 14 years made up 10.9% of examined residents and 48.4% were over fourteen years of age. The population distribution of the sample is shown in Figure 3. Mean age of the sample population was 18.8 (standard deviation [SD] 0.32) years. Females comprised 53.6% of our sample and 61.8% of participants over fourteen years of age. Among the 270 registered people who were not available for examination, 44.2% were males over fourteen years of age. However, there was no statistically significant difference between age and gender distributions of the examined population and the total population registered.

### Trachoma prevalence

**Figure 4:**
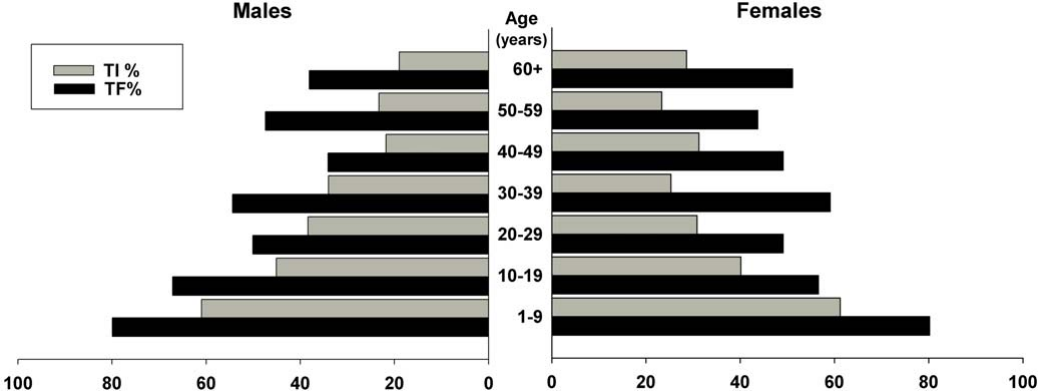
Age-specific Prevalence of Active Trachoma (TF and TI), by sex.

**Figure 5:**
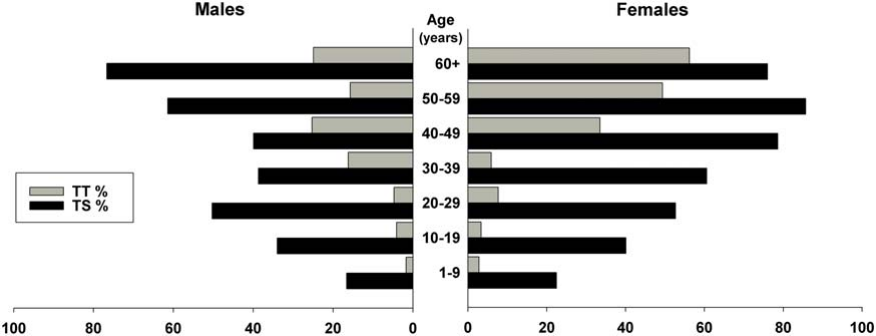
Age-specific Prevalence of Cicatricial Trachoma (TS and TT), by sex.

The prevalence of clinical signs of trachoma are shown in Table 2 while Figure 4 and 5 show the age specific prevalence of trachoma by gender. TF prevalence in children 1–9 years of age was 80.1% (95%CI, 73.9–86.3). The design effect for TF in this age group was 4.4. TI was observed in 60.7% (95% CI, 54.6–66.8) of 1–9 year-olds and either sign of active trachoma (TF and/or TI) was found in 88.3% (95% CI, 83.7–92.9). Active trachoma was found in at least one resident of 384 out of the 392 (98.0%) households surveyed.

**Table 2:** Prevalence Estimates of the Clinical Signs of Trachoma by Age group, Ayod County, Southern Sudan 2006.

Clinical Sign†	Age <1 year		Age 1–9 years		Age 10–14 years		Age >14 years	
	*n*	*Estimated %*	*n*	*Estimated %*	*n*	*Estimated %*	*n*	*Estimated %*
TF	66	67.3 (51.9–82.7)	686	80.1 (73.9–86.3)	177	67.3 (54.9–79.7)	484	50.6 (42.3–58.9)
TI	48	50.1 (34.3–65.9)	481	60.7 (54.6–66.8)	104	44.3 (36.3–52.4)	295	31.0 (23.6–38.4)
TF and/or TI	75	74.9 (63.5–86.3)	747	88.3 (83.7–92.9)	190	72.6 (61.3–83.8)	586	59.1 (50.7–67.4)
TS	6	10.2 (0.0–28.5)	148	19.3 (15.7–23.0)	84	31.7 (25.4–37.9)	630	56.5 (50.5–62.4)
TT	0	-	17	2.2 (0.73–3.6)	10	5.2 (0.0–11.3)	156	14.6 (10.9–18.3)
CO*	0	-	7	0.73 (0.0–1.7)	3	1.1 (0.0–2.7)	76	6.4 (4.5–8.3)
CO* both eyes	0	-	2	0.30 (0.0–0.78)	2	1.0 (0.0–2.6)	54	4.7 (3.4–6.0)

95% confidence limits are in ( ).

**†:** Signs may occur in combination, survey participants with multiple trachoma signs appear more than once in the table.

*: Only participants presenting with CO in the presence of TT were considered to have trachomatous CO.

The prevalence of trichiasis in children 1–14 years of age was 2.9% (95% CI, 0.4–5.3). The prevalence of TT in adults over 14 years of age was 14.6% (95% CI, 10.9–18.3). The design effect for TT in this age group was 2.7. The overall prevalence of TT was 8.4% (95%CI, 5.5–11.3). In the 392 households surveyed, one or more persons had trichiasis in 130 households (33.2%).

There were no statistically significant differences in the odds of clinical signs of trachoma between genders. The odds of trichiasis in females compared to males (all ages) was 1.79 (95% CI, 0.78–4.1), while the odds of active trachoma in girls compared to boys (age 1–9 years) was OR = 0.82 (95% CI, 0.29–2.3).

The signs of active trachoma, TF and TI, were observed most frequently in children 1–9 years of age regardless of gender. Signs of active trachoma decreased with age in both genders. Scarring was observed in young children, and was a more common finding in older age groups. TT was observed in all age groups except in children less than one year old.

### Household and Individual Characteristics

Household and individual characteristics in Ayod are displayed in Table 3. The primary source of water for 22.0% (95% CI, 14.2–29.8) of households was from an improved source. Water could be collected within 30 minutes round trip for 60.2% (95% CI 50.6–69.8) of households. The presence of a household latrine was rare; observed in 4.4% of surveyed households. Cattle were kept within 20 meters of the living quarters in 53.1% of households owning cattle. The majority (83.9%) of households in Ayod did not own a radio. Individual care givers of children reported never washing their children's faces in 23.2% (95% CI, 10.3–36.2) of households. Washing faces once per day was reported by 36.4% (95% CI, 27.8–45.0) and at least twice per day by 40.3% (95% CI, 22.4–58.3) of care givers in surveyed households. Clean faces were observed in 11.4% of children 1–9 years of age.

**Table 3:** Household and Individual Characteristics.

	Characteristic	Percent	95% CI
*Household*	Unimproved primary source of water	78.0	70.1–85.8
	*pond, river or dam*		
	Time to fetch water	39.8	30.2–49.3
	*more than 30 minutes*		
	Latrine Absent	95.6	92.3–98.9
	Solid waste disposal	79.0	63.7–94.3
	*less than 20 meters from house*		
	Cattle ownership	77.9	72.8–83.0
	Cattle kept	53.1	45.9–60.3
	*less than 20 meters from house*		
	Radio ownership	16.1	8.1–24.0
*Individual*	Never wash children's faces	23.2	10.3–36.2
	Unclean faces in children 1–9 yrs of age	88.6	83.0–94.1

### Projected targets for SAFE implementation

The projected SAFE intervention targets are summarized in Table 4. Based on the prevalence of TT found in this survey, we estimate that the total number of persons with trichiasis in need of corrective eye-lid surgery is 5,080 (lower bound = 3,580, upper bound = 7,910). Approximately 931 (lower bound = 180, upper bound = 1733) of these cases are children younger than 15 years of age. To meet the WHO recommendations, antibiotics should be distributed to all 70,000 residents in Ayod County and all 202 villages should be targeted for health education to promote facial hygiene. We estimated that the total number of households in Ayod was 10,000 (70,000 divided by the mean household size rounded to the nearest thousand). Approximately 9,560 (lower bound = 9,230, upper bound = 9,890) latrines need be built to achieve 100% household latrine coverage. The estimated number of households in need of access to an improved source of water ranges from 7,010 to 8,580.

**Table 4:** Summary of SAFE Intervention targets.

	Intervention	Estimated Need	Lower bound*	Upper bound*
S	*corrective eye-lid surgery*	5,080 persons	3,580	7,910
A	*Mass antibiotic distribution*	70,000 persons		
F	*Promotion of facial hygiene*	202 (all villages)		
E	*Access to improved water source*	7,800 households	7,010	8,580
	*Latrines*	9,560 households	9,230	9,890

*: where applicable.

## Discussion

Communities in Ayod are in urgent need of interventions to eliminate trachoma as a public health problem. Active trachoma affected nearly every household in this survey and one in three had at least one person with trichiasis. The prevalence of active trachoma in children 1–9 years of age reported in this survey is, to our knowledge, the highest district-wide estimate ever reported in Southern Sudan. Ngondi *et al* reported TF prevalence in children as high as 77.2% in a separate county within Jonglei State and TF greater than 60% in counties within two other states.[8] In addition, active trachoma in Ayod is among the highest reported globally, comparative to other trachoma hyperendemic settings in Tanzania[28],[29], Ethiopia[22],[30],[31], Kenya[32] and parts of Australia.[33],[34] Including Ayod, surveys in 12 other areas in Southern Sudan have consistently demonstrated the unreasonably high burden of trachoma (Figure 1).

The survey was not designed to capture the reasons as to why trachoma prevalence is so high in Ayod. Potential risk factors for trachoma which are common in Ayod are also seen in other counties within Sudan and in other countries. Because no samples of the organism were collected, it is not possible to compare the virulence of the organism with others found elsewhere. Additionally, it is possible that there is a unique host response because all of the people examined in this survey were of the Nuer ethnic group. The population in Ayod may be marginalized beyond that of populations elsewhere having persevered through many years of civil conflict. Access to essential medications or any health care has been almost nonexistent due to the difficulty in reaching the area. Future trachoma surveys in this area and surrounding areas should explore additional factors that may contribute to the understanding of the severity of trachoma observed.

The limitations of sampling methodology used in previous surveys have been documented.[35] The Ayod survey improves on these limitations in the use of sketch mapping and segmentation that allowed the calculation of selection probabilities and thus the ability to use sampling weights in the analysis. Sketch mapping and segmentation generally took less than forty minutes to complete and were understood and tolerated by village leaders. However, villages that covered large areas, regardless of the number of households, were more difficult to sketch and took more time. In addition, because households within each cluster had equal probability of selection, there was minimal chance of over sampling of trichiasis patients. Selected villages and segments had no prior information of our survey teams' arrival. Only the village head and a local guide were aware of the selected households once the selection of segments was complete. Survey teams walked to the selected households and enumerated only residents of those households. This removed the opportunity for blind persons to present themselves as residents of the sampled households.

In past times of conflict, drawing maps was not well accepted because of the potential security threat. This survey was limited only to areas that were considered accessible. Inaccessibility was defined on the basis of security, with localities undergoing disarmament being considered particularly risky for the safety of the survey team. There was no systematic difference in environmental conditions or ethnicity between accessible and inaccessible areas and this should not have affected the findings. Adult men were more likely to be missed at the household visit and may have introduced an over-estimation of clinical findings in adults. However, the demographics of our examined population did not differ statistically from the total registered population.

Solomon et al concluded from a study in Ghana that trained community health volunteers may be used to diagnose active trachoma and distribute antibiotics. [36] We are confident in the findings of the trained examiners in this survey. There are, however, inherent limitations in the use of the simplified grading system. The system has two grades for ‘active trachoma’ (TF and TI) which require presentation of five or more follicles greater than 0.5 mm in the central area and greater than 50% of the underlying blood vessels obscured by inflammation, respectively, in order to satisfy the grade definition. Determining the grade for each survey participant is based entirely on the observations of the grader and no verifiable evidence, such as a photograph is required. Potential sources of bias come from the spectrum of signs routinely seen by graders, and poor training. Where trachoma is severe, graders tend to under-diagnose both TF and TI since they look for unequivocal signs. Conversely, in locations where trachoma is rare, follicles that are small or few in number and moderate inflammation are often graded as TF or TI. Recognizing that the grading system has limitations does not argue for the routine use of nucleic acid amplification techniques to detect ocular Chlamydia as such tests are also subject to sources of bias at every stage from specimen collection and handling, the carriage and storage of specimens, and laboratory processing, in addition to the prohibitive costs. Now that digital photography is accessible, which does not have the same limitations as film [37], verifiable evidence of grading from digital images of the everted tarsal conjunctiva may be desirable.

Findings from this survey support anecdotal findings that the village chiefs expressed trachoma to be the most important health problem that troubles their communities. Blinding trachoma is not only severe in the adult population, but also among the children of Ayod. Trichiasis in children 1–14 years of age was nearly 30 times the WHO threshold for an acceptable level of TT cases in adults (2.9% compared to the acceptable threshold of 0.1%).[5] Up to 1,733 children in Ayod less than 15 years are in immediate need of eye-lid surgery. Without surgery, these children will likely become a burden to their families; unable to contribute to vital household duties such as fetching water, cooking, farming and cattle keeping. In addition, trachoma may have taken away the chance for marriage in children with trichiasis. At the time of the survey, two resident health care workers had received training to conduct TT surgery but no surgical kits were available for sustained activity. The need for sustained surgical intervention in Ayod is urgent and priority should be given to young children suffering from trichiasis.

According to the WHO recommendations, antibiotic distribution is needed in the entire County of Ayod to reduce current infections and community load of *Chlamydia trachomatis*. While the antibiotics are donated, the distribution costs are high. The national trachoma control program should identify funding and aim for high distribution coverage for a minimum of three years. Transmission of trachoma may be reduced in Ayod if communities adopt the behavior of face washing and proper disposal of human waste. Programs intending to initiate and encourage this behavior should identify appropriate avenues of communication. Given the low proportion of households that owned a radio, radio jingles or talk shows may not work well in this setting. The Millennium Development Goal 7 aims to reduce by half the population without access to water and sanitation.[38] Achieving this goal in Ayod would require the construction of nearly 5,000 household latrines and the provision of improved water sources to serve the same number of households. Increasing access to improved water sources may encourage the use of safe water for drinking and possibly reduce guinea worm transmission as a collateral benefit.

Finally, this survey represents an integrated activity of two neglected tropical disease programs. An extensive work-force of guinea worm surveillance field officers were trained in trachoma diagnosis, survey methodology and most importantly the SAFE strategy. Meetings with local authorities and village chiefs were used to provide education about trachoma control and explain the purpose of the survey, but also to increase awareness of guinea worm surveillance and prevention. The guinea worm eradication program was able to initiate guinea worm surveillance in areas of Ayod where surveillance did not exist and recruit new local field officers to maintain surveillance in those areas. This is one example of how disease control programs are capable of currently practicing integration. In a time of limited resources, neglected tropical disease programs should look for opportunities to integrate with others to increase the value of expenditures for otherwise single disease control activities. This core force of trained community surveillance workers will serve as the backbone of a new integrated disease elimination approach.
